# An Important Question

**Published:** 1875-04

**Authors:** 


An Important Question. — How shall we expend a fair share of our income upon food to the greatest advantage, and how shall we prepare it without destroying its nutritive properties? A savory dish of meat is often prepared by mincing or cutting the meat into small and more or less cubical blocks. It is then stewed, or more frequently boiled. The outer surface of each little block has its albumen firmly coagulated, and the whole surface is converted into about as indigestible a mass as can well be imagined—the high-priced and highly nutritious meat being destroyed for the purpose of nutrition, and the action of the digestive organs probably injured for some time to come; or good and valuable fresh meat is subjected to the process of salting, which first abstracts the juices of the meat, and then hardens the fibers so as to destroy or greatly deteriorate its digestibility. No doubt it is convenient to have a hardened, dry mass of meat, incapable of much change for months, and ready to be used for the purpose of filling the stomach and effectually satisfying the appetite; but these are not the purposes for which food was intended to be used. It ought to be capable of supplying the waste of the body, and of being easily converted into heat and motion. If it fails in these particulars, it will also fail in nourishing the brain and aiding in the evolution of intelligence; and thus intellectual and bodily power is lost to the community, and deterioration of the race is promoted.

---

Haste trips up its own heels. Don't give up a small business till you see that a large one will pay you better.

